# Silent Polymorphisms: Can the tRNA Population Explain Changes in Protein Properties?

**DOI:** 10.3390/life6010009

**Published:** 2016-02-17

**Authors:** Tamara Fernández-Calero, Florencia Cabrera-Cabrera, Ricardo Ehrlich, Mónica Marín

**Affiliations:** 1 Biochemistry-Molecular Biology, Facultad de Ciencias, Universidad de la República, Iguá 4225, Montevideo 11400, Uruguay; florenciacabreracabrera@gmail.com (F.C.-C.); ehrlich@fcien.edu.uy (R.E.); marin@fcien.edu.uy (M.M.); 2 Bioinformatics Unit, Institut Pasteur Montevideo, Mataojo 2020, Montevideo 11400, Uruguay; 3 Institut Pasteur Montevideo, Mataojo 2020, Montevideo 11400, Uruguay; ehrlich@pasteur.edu.uy

**Keywords:** synonymous polymorphisms, estrogen receptor alpha, isoacceptor tRNAs, translation kinetics, protein folding

## Abstract

Silent mutations are being intensively studied. We previously showed that the estrogen receptor alpha Ala87’s synonymous polymorphism affects its functional properties. Whereas a link has been clearly established between the effect of silent mutations, tRNA abundance and protein folding in prokaryotes, this connection remains controversial in eukaryotic systems. Although a synonymous polymorphism can affect mRNA structure or the interaction with specific ligands, it seems that the relative frequencies of isoacceptor tRNAs could play a key role in the protein-folding process, possibly through modulation of translation kinetics. Conformational changes could be subtle but enough to cause alterations in solubility, proteolysis profiles, functional parameters or intracellular targeting. Interestingly, recent advances describe dramatic changes in the tRNA population associated with proliferation, differentiation or response to chemical, physical or biological stress. In addition, several reports reveal changes in tRNAs’ posttranscriptional modifications in different physiological or pathological conditions. In consequence, since changes in the cell state imply quantitative and/or qualitative changes in the tRNA pool, they could increase the likelihood of protein conformational variants, related to a particular codon usage during translation, with consequences of diverse significance. These observations emphasize the importance of genetic code flexibility in the co-translational protein-folding process.

## 1. Introduction

Nucleotide polymorphisms are DNA sequence variations that occur frequently within a population. Silent polymorphisms (those that do not change the amino acid in the encoded protein) have only in the last decade attracted increasing attention [1]. This kind of polymorphism can produce different effects on gene expression and lead to functional differences of diverse significance. Several recent reviews summarize the effects of such mutations, in particular in relation to human diseases, personalized biomedicine and pharmacogenomics [2,3,4,5,6].

Silent mutations can affect gene expression acting at different levels and through different mechanisms. They can influence binding of regulatory factors on DNA, mRNA secondary structure and stability, ribosome traffic on mRNA and its interactions with specific ligands (as in riboswitches), including other RNAs and proteins. Silent mutations can also modify splicing, altering intron-exon boundaries or regulatory sites located in exons, enhancers or silencers, in all cases leading to an incorrect processing of mRNA. Finally, silent mutations can affect translational kinetics and protein folding, by changing codons read by tRNAs of different cellular availability. Therefore, through several mechanisms, silent mutations can give rise to differences in mRNA and protein abundance and in the structure and functionality of proteins (for a review see [5]).

In this context, we have focused on the folding and biological activity of the human estrogen receptor alpha (hERα) in an attempt to understand whether silent mutations have any effect on the functional activity of this protein. Nearly 50 synonymous polymorphisms can be found in the hERα coding sequence [7]. Some of these have been further studied in order to establish an association with different human pathologies, most of them being associated with the risk of cancer development. Additionally, evidence points towards a role for some of these synonymous variants in sperm production and cognitive disorders. Two variants called PvuII and XbaI located in intron 1, or the (TA)n repeats in the 5’ UTR, are among the main polymorphisms studied in hERα [8]. These variants were found to be associated with various pathological conditions, including cardiovascular disorders, venous thromboembolism, miscarriage, and severe pre-eclampsia [9].

Recently, we studied the activity of the hERα variant ERAla87. This variant, assigned as Bst UI, is located in exon 1 and corresponds to an alanine codon change from GCG to GCC (rs746432). The mean GCC allele frequency was estimated in about 5%, varying between 0% and 10% in Asian and European populations, respectively. This synonymous variant has been studied for an association with several pathologies, and has only been associated with mood disorders, particularly in females [10].

Using transiently transfected HepG2 and HeLa cells as an experimental approach, we were able to show that the functional activity of ERAla87 differs from that of the wild-type hERα, on a cell-type-dependent manner [11]. We propose that a conformational variant could be originated upon translation of ERAla87, as a consequence of differences in translational kinetics due to the availability of tRNA species that recognize either the GCG or the GCC codon. In this paper, we review arguments that support the idea that differences in the population of tRNAs could produce subtle changes in conformation of the ERAla87 and therefore explain the functional variations observed.

## 2. Case of Study: ERαAla87 Synonymous Polymorphism

**Figure 1:**
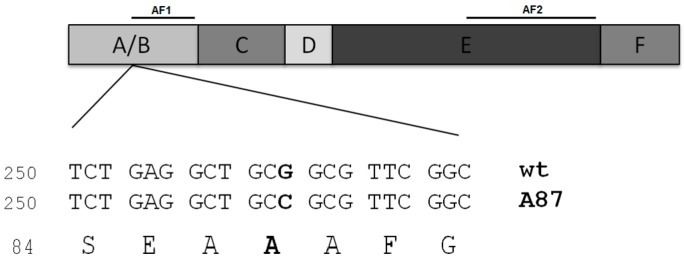
Representation of the human estrogen receptor alpha functional domains (A to F). The location of activation functions 1 and 2, AF1 and AF2, are shown (above). Below, Estrogen receptor alpha (ERWT) and silent polymorphism ERAla87 coding sequences and translated amino acids residues around Ala87 are indicated. The nucleotide change in Ala87 is shown in bold.

hERα is a transcription factor which belongs to the nuclear receptor superfamily. It mediates, together with the estrogen receptor beta (ERβ), the pleiotropic and tissue-specific effects of estrogens. hERα presents a multidomain structure (Figure 1) which includes: the A/B domain containing the activation function-1 (AF1); the C-domain which holds the DNA-binding domain (DBD); a hinge region (D-domain); an E domain which harbors the ligand-binding domain (LBD), the dimerization interface and the activation function-2 (AF2); and the carboxy-terminal domain (the F-domain) [12,13].

**Figure 2:**
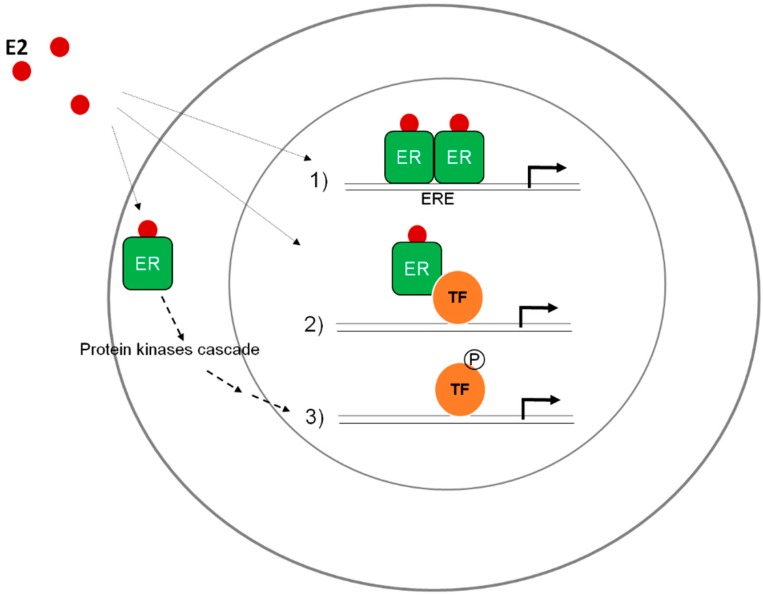
hERα mediates estrogen (E2) and other ligand effects through three main pathways. (1) The classical pathway, in which hERα binds directly to DNA, to estrogen-response elements (EREs); (2) The non-classical genomic pathway, where hERα interacts with other transcription factors (e.g., AP1 or Sp1) and regulates gene expression without directly binding to DNA; (3) The “non-genomic” mechanism, in which hERα modulates the activity of kinases that can regulate gene transcription and protein activity [19].

hERα can act through different mechanisms (Figure 2). In the classical pathway, it binds directly to DNA, specifically to estrogen-response elements (EREs) located in promoters of estrogen-responsive genes [14]. In the non-classical genomic pathway, hERα interacts with other transcription factors (such as AP1 or Sp1) and regulates gene expression without directly binding to DNA [15,16,17,18]. hERα also acts via a “non-genomic” mechanism, in which it modulates the activity of kinases that can regulate gene transcription and the activity of other proteins [19,20].

Importantly, the hERα activation mechanism, the respective contributions of AF1 and AF2 towards its activity and the agonist/antagonist effect of different ligands and promoters are all cell-specific and depend on the differentiation stage of the cell [17,18,21,22,23]. For instance, HeLa cells, originated from a cervix carcinoma, present a poorly differentiated phenotype with a cell context strictly permissive to the AF2 transactivation function of hERα. In contrast, the hepatocarcinoma HepG2 cell line shows a more differentiated phenotype, and AF1 is the dominant transactivation function involved in hERα transcriptional activity in these cells [21,24]. Though important efforts have been made to understand hERα cell-specific activity and relevant advances have already been accomplished, to date the mechanisms underlying the modulation of its activity remain elusive.

### 2.1. Differential Functional Properties of ERAla87 in HeLa and HepG2 Cells

To explore whether the ERAla87 synonymous polymorphism presents a behavior similar to that of ERWT, we analyzed its transcriptional activity and subcellular localization comparatively to ERWT, in transfected HeLa and HepG2 cell lines. For this purpose, cells were transfected with plasmids including the respective coding sequences, reporter genes, and a construct for normalization [11]. We showed that the ERAla87 transactivation activity is reduced in the classical pathway when acting through the ERE-Thymidine kinase promoter, but does not seem to be affected when acting through human complement C3 promoter (also containing ERE elements) [25]. On the other hand, ERAla87 transactivation activity is increased in the non-classical pathway when acting through AP-1 promoter and is induced by 4-OHT or ICI, which have been previously described as potent agonists on this pathway [16,17,18,23]. Finally, no significant differences were observed between the receptors in their ability to mediate non-genomic rapid effects in HeLa cells. Furthermore, by *in situ* immunofluorescence, we showed differences in the subcellular distribution of ERAla87 compared to ERWT when expressed in HeLa cells. Surprisingly, no differences in the subcellular localization were observed between ERAla87 and ERWT in HepG2 cells [11]. In brief, ERAla87 activity depends on the activation mechanism but also on the specific pathway involved within this mechanism. ERAla87 activity can be increased, decreased or remain unchanged comparatively to ERWT. Additionally, the mutation affects the subcellular localization of ER in a cell-type specific manner. How can the differences of ERαA87 functional properties be explained? 

As previously mentioned, there are several mechanisms by which synonymous mutations could affect protein activity. The experimental strategy employed bypassed the effects of synonymous variants on DNA-binding factors or on splicing. Moreover, there are no significant differences in the receptor expression at mRNA and protein levels [11]. Using RNAsnp software [26,27] to determine local RNA secondary structure changes induced by single nucleotide polymorphism (SNP), no significant differences were estimated between ERWT and ERαA87 mRNAs (not shown). Differences in miRNA and other non-coding hybridization sites as well as transcription factor-binding sites were assessed using RegRNA 2.0 software [28] and no differences were found either (data not shown)

### 2.2. Codons and tRNAs Involved in Decoding Alanine 87

Interestingly, Ala87 is the central alanine of a group of three consecutive alanines. Figure 1 shows the three consecutive alanine codons within the coding sequence. In ERWT, the three codons are GCT GCG GCG; in ERAla87, they become GCT GCC GCG. According to the human codon usage reported in the Genomic tRNA Database [29], these three codons are used as follows: GCT: 1.84%, GCG: 0.74% and GCC: 2.77%. This implies that in ERWT a local repetition of the less-used codons occurs (Ala87 and Ala88), whereas in ERAla87, one of the less-used codons is substituted by the most frequently used, also eliminating the codon repetition. (Number of genes and codon usage for alanine codons in human cells is shown in Table 1a).

Looking at the tRNAs involved in decoding alanine codons (see Table 1b,c) [30,34,35], it is interesting to note the differences in their ability to recognize the GCG codon present in ERWT or the GCC codon present in ERAla87. In fact, tRNA^Ala^-AGC, without posttranscriptional modifications in its anticodon, is able to recognize both codons; meanwhile, when adenine in the anticodon is converted to inosine, tRNA^Ala^-IGC is only capable of recognizing the GCC codon. Therefore, there will be at least the same or even more tRNA molecules capable of decoding the GCC than the GCG codon from the tRNA^Ala^- I/AGC population that is available for translation. Also, in this sense, tRNA^Ala^-GGC can only decode Ala87 (GCC). On the other hand, tRNA^Ala^-CGC and tRNA^Ala^-UGC only decode efficiently the GCG codon. tRNA^Ala^-UGC could also decode the GCC codon through wobble pairing but with less affinity. Therefore, tRNA^Ala^-CGC and tRNA^Ala^-UGC population will contribute mostly to decoding GCG codon. Taking this information into account, could the change of tRNA^Ala^ isoacceptor required to read a GCC instead of GCG be related to differences in translational kinetics and folding between ERAla87 and ERWT? In the following sections, elements that support this hypothesis are reviewed.

## 3. tRNA Abundance Can Affect Translation Kinetics and Protein Conformation

To acquire a tridimensional conformation, nascent polypeptides can follow diverse folding pathways. Sometimes folding is essentially posttranslational as it is the case for proteins which are targets of Hsp60 chaperone, and their folding occurs inside the cage formed by its 14 subunits. Many times, however, polypeptides fold during translation, while still bound to ribosomes, sequentially from the N-terminal end of the protein [36]. In this context, the kinetics of translation becomes critical since differences in the kinetics may lead to differences in folding pathways and therefore in the conformation adopted by specific proteins [37]. tRNA abundance has been proposed as a major determinant in translation kinetics, but it remains to be precisely determined in most organisms. Additionally, thermodynamic parameters of anticodon-codon recognition, which depend on the specific codon sequence, the wobble alternatives and the presence of modified bases in the anticodon loop, are relevant factors in local translation rate [38,39].

### 3.1. Role of tRNAs in the Conformation of Proteins in Prokaryotes

Codon usage bias is thought to result from selection for efficient and accurate translation of highly expressed genes [40]. In *E. coli*, in which tRNA abundance is well known, more abundant isoacceptor tRNAs were shown to correspond to more frequently used synonymous codons, decoding highly expressed proteins [41]. In this way, the use of abundant tRNAs in the synthesis of highly expressed proteins ensures higher yield and quality, by increasing translation efficiency and reducing codon misreading or aborted products. This fact has been extensively verified by  experimental approaches aiming to improve the production of recombinant proteins in bacterial systems: the overproduction of less abundant tRNAs in the expression host, or the substitution of rare codons by frequent ones in the coding sequence, can lead to a significant yield increase [42].

However, also frequently, after rare codon substitution, the increased yield of the recombinant protein in *E. coli* is accompanied by a reduction of its solubility, and its accumulation in inclusion bodies [43]. The decreased solubility suggests that a conformational change is generated by the modification of codon usage, and this can occur following either extensive changes or just a few substitutions in specific locations within the mRNA [44,45].

Indeed, rare codons have been found preferentially located in particular regions: encoding the N-terminal end of the protein, in turns or links between secondary structured regions, in links between consecutive domains or encoding signal peptides in proteins to be secreted [46,47,48]. A few works serve as illustrative examples: a study performed on the expression of EgFABP, a small fatty acid-binding protein from *E. granulosus* in *E. coli*, in which rare codons were substituted by frequent ones at a turn between two alpha helices, revealed that a synonymous variant increased its insolubility, and about 30% of the protein was detected in the insoluble fraction. The expression of the same variant triggered the activity of a heat shock promoter, indicating the presence of unfolded or misfolded proteins associated with the expression of this variant [44]. More recently, the effect of discontinuous translation at specific locations within the mRNA was analyzed on the folding of the multi-domain protein Suf1 in *E. coli*. Four slow translating regions were theoretically identified in Suf1 mRNA and their effect was analyzed experimentally. Both the addition of low-abundant tRNAs in *E coli* or the substitution of rare codons by frequent ones led to changes in the proteolysis profile, or in folding intermediates [49]. As a final example, the protein domains of epoxide hydrolases were delineated according to structural data determined for other members of the protein family. Rare codons were introduced at sites encoding links between domains, and this substitution allowed a significant increase in the solubility of the protein expressed in *E. coli* [45], indicating a role of rare codons in translation kinetics and protein conformation.

It is worth mentioning that the effect of tRNA abundance or codon usage on protein conformation has been mainly characterized for specific proteins. However, the impact of the ribosomal speed on the folding and solubility on a global, cell-wide level was addressed recently by upregulating three low-abundant tRNAs in *E. coli*. Interestingly, this upregulation led to an increased aggregation propensity of several cellular proteins and to a decreased solubility of some chaperones [50]. On the other hand, the expression of heterologous proteins in *E. coli* strains that overexpress rare tRNAs showed an increase in the insolubility of many proteins, which appears to be related to the rare codon content in the corresponding coding sequences [43].

Finally, in order to better understand the role of translation kinetics on protein folding, interesting mathematical models have been proposed, and are expected to contribute further to the knowledge of the mechanisms involved in *in vivo* protein folding [36,51,52].

Taken together, evidence so far clearly indicates that the modulation of translation dynamics in prokaryotes in relation to tRNA abundance and the choice of synonymous codons plays a critical role in a number of processes including ribosomal traffic, protein abundance, topogenesis, protein solubility and folding.

### 3.2. tRNAs, Codon Usage and Protein Conformation in Eukaryotes

In eukaryotes, the link between tRNAs, codon usage and the conformation of proteins is much less clear. In *Saccharomyces* and *Neurospora*, for example, different approaches evidence a relation between codon usage, RNA structures and protein activity. In *Neurospora*, a genome-wide correlation between codon choice and predicted secondary protein structures was observed, in which non-optimal codons appear to preferentially encode intrinsically disordered regions. This observation was verified experimentally, on the circadian clock gene *frequency* (frq), in which the change of synonymous codons affected its function *in vivo* [53].

On multicellular eukaryotes, few reports describe the effect of synonymous codon changes on protein conformation. As such variants can modify gene expression at different levels, a link with protein folding is not evident. In this sense, the study of synonymous polymorphisms in the MDR1 gene, one of the major drug transporters in human, is particularly relevant. P-glycoprotein (P-gp) encoded by MDR1 is involved in cellular expulsion of diverse compounds and in multidrug-resistance cancer cells. P-gp encoded by MDR1 carrying synonymous SNPs from a common haplotype was expressed in stably transfected polarized epithelial cells. The P-gp synonymous variants were properly synthesized and located on the cell surface, showing drug transporter activity. Interestingly, however, two of the synonymous SNPs significantly affected the stability and overall folding of P-gp. As a result, P-gp conformational alterations affected protein activity, in particular the interaction with some ligands, therefore leading to altered cellular cytotoxicity [54]. This study on MDR1 variants strongly contributed to the notion that synonymous polymorphisms can indeed affect functional properties of proteins in mammalian cells [5] and therefore are particularly relevant in the fields of biomedicine and pharmacology [55].

In general, the relevance of synonymous mutations in higher eukaryotes is mainly recognized for their association to diseases. In this context, the first identified silent mutations were shown to affect the normal splicing pattern and, more recently, as previously mentioned, other effects have also been described [5,6].

In order to understand whether synonymous variants can be related to translation kinetics and protein folding, and in turn are associated with diseases, global genomic or transcriptomic approaches are being performed. Mainly related to cancer or other complex diseases, these approaches aim to address two main concerns: (1) in relation to translation kinetics, the identification of sites of higher ribosome permanence on coding sequences during translation; and (2) the presence of synonymous variants involved in or associated with diseases. The former refers to the ribosome profiling approach which allows the identification of sites where ribosomes stay longer, thus reflecting a real traffic chart of the translational process [56]. The second consists in massive sequencing of many thousands of exomes of specific diseases and the identification of genetic variants significantly connected to the pathology [57].

The translational discontinuity revealed by ribosome profiling is a powerful experimental approach but the interpretation of results is still controversial, and from these approaches a direct relation of codon usage and translation kinetics remains unclear [58,59,60,61,62]. An important work is in progress, devoted to deciphering the biological significance of translational discontinuity, through biochemical, biophysical, genetic, cellular, and *in silico* studies [59,62,63,64]. On the other hand, massive sequencing of tumoral exomes led to the conclusion that synonymous variants can be involved in cancer and most of them appear located in oncogenes and are related to aberrant splicing [65]. However, most silent variants found in tumor suppressor p53 do not seem to be involved in the tumoral process [65]. Additionally, sequencing of melanoma cells allowed the identification of a synonymous variant F17F in the BCL2L12 gene which affects the binding of a miRNA (hsa-miR-671-5p) and the subsequent stabilization of an oncogene. Taken together, the aforementioned approaches provide valuable information about the translation process and on the identification of (synonymous) genetic variants involved in diseases. A deeper and more detailed analysis should provide clues linking silent polymorphisms with translation kinetics and protein conformation properties.

The aforementioned observations from eukaryotes suggest that conformational changes due to modified translation kinetics could account for functional differences, such as those found in ERAla87. Considering this, we wondered whether the change of isoacceptor tRNA^Ala^ required to read a GCC instead of GCG codon may be related to a modification of local translation kinetics of the receptor mRNA. In general, the poor knowledge on tRNA abundance in higher eukaryotes has been an obstacle for the estimation of translation kinetics based on tRNAs’ relative concentration. How to evaluate the tRNA population?

## 4. Dynamic Populations of tRNAs

As has already been mentioned, a general fit of the translational system was highlighted through a strong correlation between the frequency of synonymous codon usage and the corresponding population of decoder tRNAs’ molecules in the cell. For example, early reports on cells with extremely biased protein expression revealed high frequencies in the use of selected codons and high concentrations of the specific decoder tRNAs. This was the case for isoaccepting tRNA^Ala^ and tRNAS^Ser^ species present in the posterior gland of the silkworm *Bombyx mori*, described several decades ago [66,67,68].

Nevertheless, although in bacteria and yeast it was clearly shown that the abundance of tRNA isoacceptors correlates with the codon usage of abundant proteins, in metazoans this correlation appeared less strict [69]. In this case, which is the real correlation between the tRNA population and codon usage? Is it the same in unicellular organisms and metazoans? Beyond classical works reporting differential expression of tRNA genes (for instance, tRNAs present in the gland of the aforementioned *Bombyx mori*), the question remains if the tRNA population is tissue- or cell-specific. Does the tRNA population of a given cell change during cell cycle?

### 4.1. tRNA Genes Are Differentially Expressed in Different Cell States

Over the last few years, different lines of research based on holistic approaches, progressively converged, and began to shed more light on the biological roles of tRNA. Notably, through characterization (although partial) of the tRNA population by deep sequencing [70], microarrays [71,72] and chromatin analysis at the tRNA loci [71], it appears that tRNA genes are in fact differentially regulated. In this context, it has been described in *S. cerevisiae* that specific changes in tRNAs’ copy number were associated with specific stress responses [70]. Moreover, after a semi-quantification of tRNA pools in different human cells, the existence of two distinct translation programs that operate during proliferation and differentiation was proposed [71]. Furthermore, it was shown that alterations in the tRNA repertoire of proliferating and differentiated cells correspond to codon usage preferences of proliferation- or differentiation-regulated genes as revealed by transcriptomic studies, measurement of tRNA pools, gene ontology analysis that groups functionally related genes, and the analysis of active chromatin and RNA polymerase III occupancy at the level of tRNA genes [71]. This indicates that modification of the levels of specific tRNAs is concerted with changes in the transcriptome, in order to optimize codon usage of genes that are being expressed. Among other works supporting this view, it is worth mentioning that significant differences in tRNA composition have been found between breast cancer cells and non-transformed tissue, suggesting an adjustment of tRNA pools in cancer cells adapted to translate mRNAs associated with tumor progression [73]. These changes in tRNA repertoire strongly suggest an extremely precise coordination between transcription and translation in eukaryotic cells, involving fine regulatory mechanisms that ensure the adaptation of the translation apparatus to specific cell states [74].

### 4.2. tRNA Post-Transcriptional Modifications: Expanding the Complexity of the tRNA Population

Concerning the link between the tRNA population and codon usage, it is also important to consider tRNA post-transcriptional modifications which have been intensively studied over several decades [38,75,76,77]. To date, roughly 100 modified nucleosides have been found in tRNAs from bacteria, archaea and eukaryotes. tRNA modifications involve a large set of specific enzymes (methyl-transferases, transglycosylases, transferases, adenosine desaminases, pseudouridine synthases, thiouridylases, among others) present in different cell compartments, which exhibit high specificities for tRNA species, particular target bases, and the precise location in the tRNA structure (for a review see [75,78,79]).

Modified bases are required for efficient protein synthesis, stabilizing tRNA structure, ensuring specificity and stability of codon-anticodon interaction, preventing frameshift errors and participating in the specificity of aminoacyl-tRNA synthetase [76]. Concerning their involvement in codon-anticodon interaction, the role of modified (and frequently hypermodified) bases in positions 34 of the anticodon (wobble) and 37 in the anticodon loop, has been highlighted both in prokaryotes and eukaryotes. They appeared to be critical in defining kinetic and thermodynamic parameters of codon recognition and hence of the translation process [38,76,78,80].

Nevertheless, their role is not limited to the translation process. Indeed, they are critical in tRNA quality control and turnover [81], cellular localization of tRNA molecules, and are also related to tRNA fragments’ biogenesis [82]. Furthermore, changes in the profile of tRNA modification have been described as associated with genetic diseases [83,84], microbial infections [85,86], immune response [87] and cellular stress [88]. It is worth mentioning that tRNA base modifications participate in translation regulation in response to change in cellular programs, as revealed in yeast under stress conditions [89].

Therefore, it is interesting to consider that the population of each tRNA species may be further subdivided into subspecies, characterized by different base modification profiles. If the enzymatic modification process, in addition to sequential modification events, also varies depending on cellular state, then the number of subspecies of a given tRNA could be very high. Finally, it is worth mentioning that the implication of altered tRNA base modification profiles in different phenotypes and diseases has been proposed for many years. More recently, from genome-wide association studies, an important number of human pathologies, such as alterations of metabolic pathways, mitochondrial defects, neurological disorders and increased susceptibility to cancer, have been associated with mutations in genes coding for tRNAs and tRNA-modifying enzymes (for a review see [83]).

### 4.3. The tRNA Pool Is Partitioned in Different Cell Compartments

After transcription, the tRNA cycle includes different steps: maturation (processing of 5’ and 3’ trailers, intron splicing for those tRNA carrying an intervening sequence), posttranscriptional modification, precise cellular localization, interactions with partners involved in their different roles, and, finally, quality control that includes their degradation in the cytoplasm and/or the nucleus involving complex translocation mechanisms (reviewed in [90,91]).

Mature tRNAs—free or aminoacylated—interacting with molecules and molecular complexes involved in translation, can be considered as partitioned in different cell compartments: initiation and elongation factors, aminoacyl-tRNA synthetases (either free or included in multi-synthetase complexes in eukaryotes and archaea [92]), or translating ribosomes, among others. Interestingly, a “tunneling” phenomenon has been described by which tRNAs involved in translation, cycle among elongation factor eF2, aminoacyl-tRNA synthetases and translating ribosomes [92,93,94,95]. In agreement, from studies in the yeast *S. cerevisiae*, it was proposed that once a particular codon has been used, subsequent occurrences of the same amino acid do not use codons randomly, but favor codons that use the same tRNA. The reported data suggest that tRNA diffusion away from the ribosome is slower than translation [96]. Nevertheless, this important issue is still open [97].

As more evidence becomes available, the definition of the tRNA population turns more complex and its dynamic nature becomes more evident. Which subspecies of a particular tRNA is actually involved in decoding a certain codon? Is it preferentially located in a certain compartment? What are the posttranslational modifications it requires to fulfil this role? And finally, how is this regulated in order to ensure an efficient translation process, adjusted to different cell states? These issues need to be taken into account when trying to determine the relation between tRNA availability, codon usage and ultimately protein conformation and function. In this sense, the development of new techniques for an accurate measurement of the different tRNA species (or even subspecies) will be of paramount importance.

## 5. General Conclusions

### The Sound of Silent Substitutions: The Tale of the Princess and the Pea, and the Case of Synonymous Polymorphism Ala87Ala GCG->GCC in Human ERα

As an approach to understanding the basis of the regulation of hERα and the effect of synonymous polymorphisms on its activity, we previously showed functional differences exhibited by ERAla87 comparatively to ERWT in HepG2 and HeLa transfected cells. After discarding other hypotheses, in this work our aim was to review arguments for a role of a conformational change in the ER (which would lead to the described functional changes) brought about by a possible change in the identity of the tRNA decoding Ala87. Considering human codon usage, the Ala87 codon change involves the substitution of a rare codon (in WT) by a frequent one (in ERAla87). In addition, this change suppresses a codon repetition (in WT) leaving instead three consecutive different Ala codons. Looking at the involved isodecoder tRNAs^Ala^, it is interesting to note that tRNA^Ala^ carrying inosine (tRNA^Ala^-IGC) and tRNA^Ala^-GGC can only decode Ala87 (GCC). Also, they could read two consecutive codons in the silent polymorphism, though only one in the WT. On the other hand, as analyzed previously, the tRNA^Ala^-CGC and tRNA^Ala^-UGC populations would contribute mostly to decoding the WT (GCG). According to these observations it seems possible that when ERWT and ERAla87 are expressed in the same cell lines, the alanine codon change could lead to a modification of the local translation dynamics and to a change in the protein conformation.

But how can the functional activity of ERAla87 differ from that of the wild-type hERα, on a cell-type-dependent manner? As reviewed, changes in the tRNA population have been associated with proliferation, differentiation or response to chemical, physical or biological stress. These findings imply quantitative and/or qualitative differences in the tRNA pool between cell lines that could increase the likelihood of protein conformational variants, related to a particular codon usage during translation.

Nonetheless, tRNA availability is still not well understood in multicellular organisms. The lack of information about the real concentration of each tRNA under different physiological conditions hampers the evaluation of translation kinetics and hence the analysis should be considered with caution. The development of new sequencing methods, allowing the identification of modified bases in tRNAs, is urgently needed for a deeper understanding of the roles of tRNAs in the cell and the real tRNA availability for protein translation in each tissue, each cellular condition and for each subcellular compartment. This information will be crucial in order to truly determine if our hypothesis regarding the role of tRNAs in the conformation and functional properties of hERα synonymous variants is correct.

The relationship between tRNAs, codon usage and the process of co-translational protein folding is currently the subject of intense work. The convergence of holistic approaches, like deep sequencing and ribosome profiling, among others, together with biochemical and cellular approaches (with particular attention paid to the posttranscriptional modifications of RNA and its regulation, global cellular adaptation, from transcription-translation concerted regulation to the adjustment of the translational apparatus to specific cell states), will certainly provide important elements in the near future. In addition, computer modeling approaches will surely lead to powerful integrative landscapes.

Clearly, multiple interdependent factors are involved in the co-translational folding, and that fact could be the basis for divergent reports, each focusing on different aspects. For instance, several reports, based on ribosome profiling studies, have reached contradictory conclusions about the correlation between high translation elongation rates and codon frequencies and/or isoacceptor tRNA abundance [56,61]. In this sense, it was recently shown in yeast that the overexpression or deletion of tRNAs, as well as swapping of anticodons, analyzed by different approaches including ribosome profiling and kinetic modeling, did not show a direct correlation between codon adaptation and translational efficiency, describing instead a correlation between strong mRNA secondary structures and a local speed reduction of translating ribosomes [62]. Such contradictory results could arise from conceptual and/or technical issues. Conceptual aspects could be connected to the diversity of the tRNA population (isoacceptors, isodecoders, different species, different modification states, possibly different compartmental distribution). Technical aspects could be related to the precision in determining ribosomal dwell sites at the codon level. In this sense, several recent papers focusing on technical considerations about translation arrest to stabilize ribosomes for measuring their positions, strongly support that translation kinetics is linked to the concentration of decoder tRNAs [58,59,98] and to their modification state [99]. Moreover, concerted global regulatory mechanisms associated with translation demands should also be considered. This was recently highlighted through the observation that the deletion of a tRNA gene in yeast breaks the translational balance, thereby causing the tRNA pool to rapidly evolve to meet the new translational demands, suggesting—even though the evolutionary scenarios that trigger changes in the tRNA pool have yet to be thoroughly explored—that genomic duplications, deletions, and anticodon mutations could shape tRNA gene families [100].

Possibly within the large diversity of the cellular tRNA population, their complex life cycles and multiple functions, new clues will arose that will help to solve the present challenges.

In conclusion, the correlation between codon usage and tRNA population and its complexity should be subtler than a general fit, even taking into account tRNA transcription levels or their global abundance in specific cell contexts. The precise effect of tRNAs on the translation of a specific mRNA might vary depending on the cell state. It could be subtle or extensive, but in any case it could result in conformational changes which can in turn give rise to polypeptides that expose transient hydrophobic surfaces prone to aggregation, alternative proteolytic profiles, and therefore to altered functional properties. The conformation of the ERα has been proposed as a major regulator of its own activity [101] and, additionally, different conformations were detected in ERWT when synthesized in different *in vitro* translation systems by chymotrypsin-limited proteolysis [102]. All these elements lead us to consider that a subtle conformational change occurs in the synonymous variant ERAla87 which may be involved in its differential behavior.

## Figures and Tables

**Table 1:** (**a**) Number of genes and codon usage for alanine codons in human cells. (**b**) Wobble pair rules reviewed in [30]. (**c**) Analysis of ERWT and ERAla87 codon-anticodon recognition by alanine tRNAs restricted to tRNA Ala sequences described in the literature according to Modomics [31,32,33]. * indicates that recognition is not frequent in cell context. ↓ indicates low affinity recognition.

**a. Alanine tRNA Genes in Human Genome According to the Genomic tRNA Database [29]**			
Anticodon (5′ → 3′)	Corresponding Codon (5’ → 3’)	nº of Genes	Genome Codon Usage
AGC	GCT	30	1.84
GGC	GCC	1	2.77
CGC	GCG	5	0.74
UGC	GCA	10	1.58
**b. Wobble Pair Rules Reviewed in [30]**			
tRNA 5’ Anticodon Base	mRNA 3’ Codon Base	ER Ala 87 Polymorphism Recognized by tRNA	
G	U,C	Ala87	
C	G	wt	
k^2^C	A	---	
A	U,C,G>A	wt, Ala87	
U	U,A,G>C	wt, Ala87 ↓	
xm^5^s^2^U,xm^5^Um,Um,xm^5^U	A>G	wt ↓	
xo^5^U	U,A,G	wt	
I	A,C,U	Ala87	
**c. Analysis of ERWT and ERAla87 Codon-Anticodon Recognition by Alanine tRNAs Restricted to tRNA Ala Sequences Described in the Literature According to Modomics [31,32,33].**			
tRNA-AGC:	-without modifications recognizes: wt *, Ala87 *		
	-modified A **→** I recognizes: Ala87		
tRNA-CGC:	-without modifications recognizes: wt *		
tRNA-UGC:	-without modifications recognizes: wt, Ala87 ↓		
	-with modifications recognizes: wt			
tRNA-UGC:	-without modifications recognizes: Ala87		
